# The predictive value of pretreatment hemoglobin-to-platelet ratio on osteoradionecrosis incidence rates of locally advanced nasopharyngeal cancer patients managed with concurrent chemoradiotherapy

**DOI:** 10.1186/s12903-023-02937-9

**Published:** 2023-04-20

**Authors:** Busra Yilmaz, Efsun Somay, Erkan Topkan, Ahmet Kucuk, Berrin Pehlivan, Ugur Selek

**Affiliations:** 1 Department of Oral and Maxillofacial Radiology, Faculty of Dentistry, Baskent University, Ankara, Turkey; 2 Department of Oral and Maxillofacial Surgery, Faculty of Dentistry, Baskent University, Ankara, Turkey; 3 Department of Radiation Oncology, Faculty of Medicine, Baskent University, Adana, 01120 Turkey; 4 Department of Radiation Oncology, Mersin City Hospital, Mersin, Turkey; 5 Department of Radiation Oncology, Bahcesehir University, Istanbul, Turkey; 6 Department of Radiation Oncology, School of Medicine, Koc University, Istanbul, Turkey

**Keywords:** Osteoradionecrosis, Nasopharyngeal cancer, Hemoglobin-to-platelet ratio, Concurrent chemoradiotherapy, Head and neck cancer

## Abstract

**Background:**

This retrospective study aimed to investigate whether the pretreatment hemoglobin-to-platelet ratio (HPR) could predict the risk of osteoradionecrosis (ORN) in patients receiving concurrent chemoradiotherapy (C-CRT) for locally advanced nasopharyngeal carcinoma (LA-NPC).

**Methods:**

ORN cases were reported from the records of LA-NPC patients who had oral examinations before and after C-CRT. The pretreatment HPR values were calculated on the first day of C-CRT. The connection between HPR values and ORN occurrences was determined using receiver operating characteristic curve analysis. The primary endpoint was the relationship between the pretreatment HPR values and post-C-CRT ORN incidence rates, while secondary endpoints included the identification of other putative ORN risk factors.

**Results:**

We distinguished 10.9% incidences of ORN during the post-C-CRT follow-up period among 193 LA-NPC patients. The optimal cutoff for pre-C-CRT HPR was 0.48 that grouped the patients into two HPR groups with fundamentally different post-C-CRT ORN incidence rates: Group 1: HPR ≤ 0.48 (N = 60), and Group 2: HPR > 0.48 (N = 133). The comparative analysis indicated a significantly higher ORN incidence in HPR ≤ 0.48 group (30%; P < 0.001). The other factors associated with meaningfully increased ORN rates included the presence of pre-C-CRT ≥ 5 teeth extractions, mandibular volume receiving ≥ 64 Gy, post-C-CRT tooth extractions, mean mandibular dose ≥ 50.6 Gy, and C-CRT to tooth extraction interval > 5.5 months.

**Conclusion:**

Low pretreatment HPR levels were independently and unequivocally linked to significantly increased incidence of ORN post-C-CRT. Pre-C-CRT HPR levels may be used to estimate the incidence of ORN and be useful for taking preventive and therapeutic measures in these patients such as monitoring oral hygiene with strict follow-up, avoidance of unnecessary tooth extractions, particularly after C-CRT, and use of more rigorous mandibular RT dose limits.

**Supplementary Information:**

The online version contains supplementary material available at 10.1186/s12903-023-02937-9.

## Background

Concurrent chemoradiotherapy (C-CRT) is currently considered the gold standard treatment for locally advanced nasopharyngeal cancers (LA-NPC) [1]. Sadly, this treatment was accompanied by an increase in early complications like mucositis and taste abnormalities as well as an increase in late complications like radiation-induced trismus, dysphagia, and corresponding declines in quality of life measures [2, 3]. Another serious and occasionally life-threatening complication of C-CRT is osteoradionecrosis (ORN), which is more likely in particular individuals who have tooth decay and require extraction(s) [4–6]. Despite precautions, some patients are still subjected to ORN [7, 8].

Poor oral health before, during, and after C-CRT has been linked to an increased risk of ORN, as have post-radiation minimally invasive or invasive surgical procedures, such as dental implant placement and tooth extraction in the radiation field [9–11]. Historically, the incidence of ORN in such patients was estimated to be between 2% and 22% [12–15], while newer studies indicate it to be in the range of 3–7% following modern radiotherapy techniques [16, 17]. The immunosuppressive C-CRT may cause infectious deep dental caries, periodontal diseases, and periapical lesions, which are thought to be the consequence of inadequate repair mechanisms of irradiated tissues [18, 19, 20]. Furthermore, tooth extractions and poor oral hygiene significantly raise the risk of ORN by 89.3% and 71.4%, respectively [9, 21–26]. The pathophysiology of ORN is primarily characterized by the development of hypoxia, hypovascularity, and hypocellularity as a result of radiation-induced damage to healthy vascular endothelium [27]. An emerging theory in ORN pathogenesis called tissue fibrosis explains how fibroblast activation and dysregulation lead to the development of atrophic tissue at the irradiated site, the first step in ORN formation [28]. In LA-NPC patients undergoing C-CRT, the mandible, which has a poor blood supply by nature, enters the radiation field in varying magnitudes and receives high irradiation doses, making this area more prone to ORN development due to induced local hypoxia and activated fibrotic tissue repair processes [29].

A potential biomarker that represents the patient’s oxygenization status, vascular potency, inflammatory, and immunological condition, regardless of the underlying reason, is the hemoglobin-to-platelet ratio (HPR), which is a combination of hemoglobin and platelets [30]. It has been reported that in some inflammatory diseases, vascular endothelial inflammation, edema and fibrosis developing especially at the capillary level due to low hemoglobin levels may result in persistent hypoxia throughout the entire body [31]. And this hypoxic condition, which is thought to be responsible for directing the fibrosis process, may result in excessive scarring in the tissues [32]. Furthermore, platelets produce vascular occlusion by participating in primary hemostasis, hence it has been suggested that a high platelet count may promote hypoxia by interrupting blood flow at the tissue and vessel levels [33]. Stimulated hypoxia-induced factor-1 (HIF-1) and angiogenic factors such as vascular endothelial growth factor (VEGF), transforming growth factor (TGF-α and TGF-β) have been shown to play critical roles in the ORN development due to reduced vascularization and tissue hypoxia [34–36].

Previous studies have frequently proven a substantial link between the HPR and prognosis in a variety of clinical settings, including multiple cancer types and vascular diseases [30, 37–39]. However, the true significance of HPR, a biomarker that may indicate hypoxia, reduced angiogenesis, and vascular potency in tissues, in predicting the occurrence of ORN has never been investigated. As a result, we intended to retrospectively evaluate the significance of pretreatment HPR values for predicting ORN after C-CRT in LA-NPC patients who received oral care before C-CRT.

## Patients and methods

### Ethical approval

This study protocol was approved by the institutional review board of Baskent University Medical Faculty and was in compliance with the Declaration of Helsinki (No. D-KA/19–39). All patients gave informed and signed permission before beginning assessments, either themselves or legally charged caretakers for the participation, collecting and interpretation of blood samples, sociodemographic and medical records, dental X-rays, and academic presentations.

### Study population

We conducted a review audit of the institutional clinical records of LA-NPC patients who went through oral and dental exams before the C-CRT performed between January 2010 and December 2021 in the Dentistry Clinics of the Adana Research and Treatment Center at Baskent University. The TRIPOD (Transparent Reporting of a multivariable prediction model for Individual Prognosis Or Diagnosis) Statement was used for this study design. The following criteria were required to be qualified for this study: Aged ≥ 18, treated for histopathologically demonstrated squamous cell carcinoma of the nasopharynx, proven locally advanced disease per American Joint Cancer Committee (AJCC) 8th ed, no history of other cancers, not received systemic chemotherapy or RT to the head and neck region before the dental evaluations but have undergone conclusive C-CRT then, and accessible pre-C-CRT and follow-up records of panoramic radiography, and pre-C-CRT complete blood count tests. Ineligibility criteria included the presence of mandibular invasion by the local extension of the primary tumor or the metastatic lymph nodes, a history of ORN diagnosis, and steroid use in the past 30 days before the start of C-CRT. To forestall the unexpected biasing impact of baseline immune and inflammatory conditions and drug usage, patients with systemic inflammatory conditions such as rheumatological diseases, nephritic disorders, respiratory diseases, viral hepatitis, proven immune suppressive disorders, history of collagen diseases, and chronic inflammatory conditions excluded from the present analysis.

### Baseline oral examination

Even if they were asymptomatic, all patients underwent a regular oral examination before the C-CRT, as recommended by the American Dental Association (ADA) and the US Food and Drug Administration (FDA) [40]. All the clinical and radiological examinations were assessed by an experienced oral and maxillofacial radiologist (BY) and surgeon (ES). For each patient, panoramic radiographs were used to perform radiographic oral exams in accordance with our institutional norms for such patients. All digital panoramic radiographs were acquired with the same X-ray device (J Morita, Veraviewepocs 2D, Kyoto, Japan) set at 70 kVp, 10 mA, and 9 s, and patients were positioned according to the manufacturer’s recommendations.

All patients received a standard dental management program following our institutional standards for such patients. The patients were given instructions on how to maintain good oral hygiene, including using a soft toothbrush, dental floss, an interproximal brush, fluoride toothpaste, and daily topical fluoride mouthwash. The loose contact points that could lead to food impaction and the sharp tooth edges that could harm the oral mucosa were both fixed. For the teeth that could be restored, endodontic procedures were carried out. Teeth with internal or external root resorption, pulpal, periodontal, or periapical diseases, root caries covering more than half of the root circumference, fully or partially impacted teeth with follicular cysts, or teeth with residual roots were extracted [9].

### Hemoglobin-to-platelet ratio (HPR) assessments

For each patient, we calculated the pre-C-CRT HPR values [HPR = Hemoglobin level (g/dL) ÷ platelet count (× 10^3^ per µL)] using the platelet and hemoglobin measurements acquired on the first day of the C-CRT.

### Chemoradiotherapy protocol

All patients received simultaneous integrated boost intensity-modulated RT (SIB-IMRT). Pretreatment co-registered computed tomography (CT), 18-FDG-PET–CT (18-fluorodeoxyglucose-positron emission tomography/computed tomography), and/or magnetic resonance imaging scans of the implicated main site and the whole neck were used to define all target volumes. The target volumes and related RT doses were as described elsewhere [41]. In brief, according to institutional RT practice for such patients, the prescribed doses for the high-, intermediate-, and low-risk planned target volumes (PTV) were 70 Gy, 59.4 Gy, and 54 Gy delivered on a daily fractionation basis (5 days per week for 33 days), respectively. The chemotherapy protocol comprised of seven weekly doses of cisplatin (40 mg/m^2^) administered concurrently with RT.

### Follow-up oral examination

The same methodology as described in the “Baseline oral examination” section was used for all follow-up oral examinations. The treatment commitments for each patient were determined and reported using the previously stated principles. The ORN status was determined using the previously mentioned clinical and radiological ORN diagnostic criteria and stage, as well as the presence of ORN with intact mucosa on diagnostic imaging. ORN was clinically defined as irradiated necrotic bone tissue that failed to heal after 3 months without tumor progression or metastasis. Radiologically, the sequestrum representing a radiopaque necrotic bone fragment, large or localized osteolytic areas, jawbone fracture, and also radiolucent areas surrounding the tooth extraction sockets that remained visible for more than 12 months were determined [9, 35, 36, 40]. ORN staging was performed using Notani’s classification, which includes minor bone changes and anatomical boundaries of lesions [42]: Stage 1: ORN is limited to the alveolar bone; Stage 2: ORN is limited to the mandible above the alveolar bone and/or the mandibular alveolar canal; Stage 3: ORN is expanding below the mandibular alveolar canal, as well as pathological fracture and/or skin fistula.

As stated in the “Baseline oral examination” section, oral hygiene education was continued throughout the C-CRT and follow-up periods. Regular oral exams and restorative procedures were performed as needed. Endodontic procedures were carried out and, if required, loose contact points that might result in food impaction were repaired. Every effort was made to avoid invasive surgical procedures [9].

### Statistical analysis

The relationship between pre-C-CRT HPR esteems and post-C-CRT ORN rates comprised the primary endpoint. Continuous variables were described using medians and ranges, whereas categorical variables were expressed using percentage frequency distributions. The Chi-square test, Student’s t-test, or Spearman correlation analyses were employed to compare the patient groups, as indicated. The pre-C-CRT HPR cutoff that, if present, may separate the entire research cohort into two HPR groups with different results were estimated using receiver operating characteristic (ROC) curve analysis. The dosimetric parameters the median maximum mandibular point dose (MMPD) and the mean mandibular dose (MMD), which may interact with ORN rates, were measured using the Eclipse Treatment Planning System (Varian Medical Systems). A two-sided P-value of less than 0.05 was considered statistically significant.

To estimate the effect of clinical, biochemical, and dosimetric variables on ORN, a multivariate logistic regression model was constructed, which included the mandibular volume receiving (V64), MMD, the presence of pre-C-CRT or post-C-CRT any number of teeth extractions, a C-CRT to tooth extraction interval, and HPR values that were statistically significant in univariate analysis [43]. Finally, the association between HPR and ORN status was investigated using stepwise backward binary regression. V64, MMD, pre- and post-C-CRT tooth extractions, C-CRT to tooth extraction interval, and HPR values were all taken into account, with ORN status as the dependent variable. In the stepwise backward multivariate method, only the variables that were statistically significant in univariate analysis were included, while the α value for the p-value in the stepwise backward model was set to 0.157 as suggested by Heinze and Dunkler [44].

## Results

**Fig. 1:**
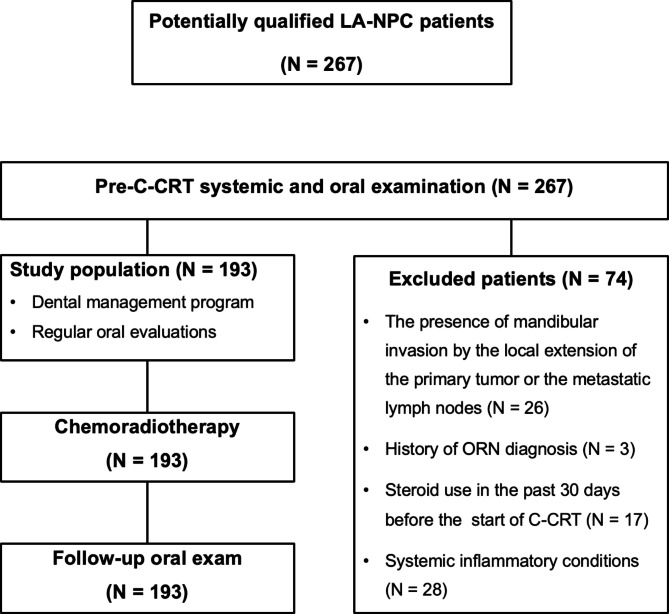
The flowchart describes the eligibility, management and follow-up status of the participants throughout the study. *Abbreviations: C-CRT* concurrent chemoradiotherapy, *LA-NPC* locally advanced nasopharyngeal carcinoma, *ORN* osteoradionecrosis

As demonstrated in Fig. 1, a total of 267 LA-NPC patients were evaluated and 74 cases were excluded from the study due to the presence of mandibular invasion by the local extension of the primary tumor or the metastatic lymph nodes (N = 26), history of ORN diagnosis (N = 3), steroid use in the past 30 days before the start of C-CRT (N = 17), and systemic inflammatory conditions (N = 28). A total of 193 LA-NPC patients who received pretreatment oral and dental evaluations before the initiation of C-CRT were eligible, according to the current database search. As displayed in Table 1, the median age was 54 years (range: 18–76), with a male gender preponderance (71.5%). Patients who had previously smoked or consumed alcohol made up 92.2% and 82.4% of the entire population, respectively. Most patients had locally and/or regionally advanced NPC, namely 75.1% and 80.8% of the study group had T3–4 and N2–3 disease stages, respectively. Reflecting their general poor oral and dental health conditions, all patients underwent at least one tooth extraction as mandated by the assessments, with a median of 3 (range: 1–11) extracted teeth before the commencement of the C-CRT. The median interval from tooth extraction to the start of C-CRT was 17 days (range: 12–27). Every patient received an unremitting dental management program.

**Table 1:** Baseline and treatment characteristics for the entire study cohort and per hemoglobin-to-platelet ratio status

Characteristic	All patients (N = 193)	HPR ≤ 0.48 (N = 60)	HPR > 0.48 (N = 133)	*P-*value
Median age, years (range)	54 (18–76)	53 (18–76)	55 (21–75)	0.94
Age group, N (%) ≤ 54 > 54	87 (45.1) 106 (54.9)	30 (50.0) 30 (50.0)	57 (42.9) 76 (57.1)	0.34
Gender, N (%) Male Female	138 (71.5) 55 (28.5)	38 (63.3) 22 (36.7)	100 (75.2) 33 (24.8)	0.12
Smoking status, N (%) Yes No	178 (92.2) 11 (7.8)	56 (93.3) 4 (6.7)	126 (94.7) 7 (5.3)	0.91
Alcohol consumption, N (%) Yes No	159 (82.4) 34 (17.6)	47 (78.3) 13 (21.7)	112 (84.2) 21 (15.8)	0.26
Periodic dental management, N (%) Absent Present	0 (0.0) 193 (100.0)	0 (0.0) 60 (100.0)	0 (0.0) 133 (100.0)	1.0
Median pre-C-CRT tooth extraction, N (%)	3 (1–11)	4 (1–11)	3 (1–7)	0.72
Pre-C-CRT tooth extraction group, N (%) ≥ 5 < 5	70 (43.0) 123 (57.0)	27 (45.0) 33 (55.0)	26 (19.5) 107 (80.5)	< 0.001
Median tooth extraction time to C-CRT, days	17 (12–27)	16 (12–26)	18 (13–27)	0.69
T-stage group, N (%) 1–2 3–4	48 (24.9) 145 (75.1)	16 (26.9) 44 (73.1)	32 (24.0) 101 (76.0)	0.63
N-stage, N (%) 0–1 2–3	37 (19.2) 156 (80.8)	12 (20.0) 48 (80.0)	25 (18.8) 108 (81.2)	0.84

*Abbreviations: C-CRT* concurrent chemoradiotherapy, *HPR* hemoglobin-to-platelet ratio, *N* node, *T* tumor

Tooth extractions were similarly prevalent throughout the post-C-CRT follow-up period, with 83.9% of patients requiring at least one extraction (range: 0–5) and a median time from C-CRT to tooth extractions of 7 months (range: 1–13). After a median follow-up of 20.8 months (range: 12.4–27.6), a total of 21 ORN cases were identified, indicating an ORN incidence of 10.9%. All ORNs manifested in the affected mandible’s posterior region and after tooth extraction, specifically in the areas with higher RT doses, with all ORNs being diagnosed in patients receiving a MMD > 41.2 Gy. The extracted teeth in 10 patients were noted as regions 35, 36, 37, and 38, and in 11 patients as regions 45, 46, 47, and 48, according to the World Dental Federation (FDI) inscription teeth numbering system (ISO 3950). As depicted in Table 2, the median MMPD for the whole research group was 62.6 Gy (range: 52.7–71.8), whereas the MMD was 48.6 Gy (range: 17.4–64.1). Comparatively, the MMD was significantly higher in the presence of ORN than in the absence of ORN group (54.7 vs. 38.9 Gy; P < 0.001), although the median MMPD values were indistinguishable (71.8 vs. 70.2 Gy; P = 0.94).

**Table 2:** The relationship between post concurrent chemoradiotherapy tooth extraction, mandibular dose, post concurrent chemoradiotherapy osteoradionecrosis, concurrent chemotherapy cycles and two hemoglobin-to-platelet ratio groups

Characteristics	All patients (N = 193)	HPR ≤ 0.48 (N = 60)	HPR > 0.48 (N = 133)	*P-*value
Median MMPD; Gy (range)	62.6 (52.7–71.8)	63.7 (53.8–70.7)	61.1 (52.7–71.8)	0.89
MMD, Gy (range)	48.6 (17.4–64.1)	47.3 (17.4–64.1)	49.9 (19.2–63.4)	0.37
MMD group, N (%) < 50.6 Gy ≥ 50.6 Gy	132 (68.4) 61 (31.6)	42 (70.0) 18 (30.0)	90 (67.7) 43 (32.3)	0.78
V64 Gy group, N (%) < 27% ≥ 27%	158 (81.9) 35 (18.1)	50 (83.3) 10 (16.7)	108 (81.2) 25 (18.8)	0.86
Continued smoking, N (%) Yes No	64 (33.2) 129 (66.8)	21 (35.0) 39 (65.0)	43 (32.3) 90 (67.7%)	0.67
Continued alcohol consumption, N (%) Yes No	56 (29.0) 137 (71.0)	17 (28.3) 43 (71.7)	39 (29.3) 94 (70.7)	0.90
Median post-C-CRT extracted teeth, N (range)	1 (0–5)	2 (0–5)	1 (0–4)	0.38
Post-C-CRT tooth extraction, N (%) Absent Present	31 (16.1) 162 (83.9)	11 (18.3) 49 (81.7)	20 (15.0) 113 (85.0)	0.87
Median time from C-CRT to tooth extraction, mo. (range)	7 (1–13)	6 (1–11)	8 (1–13)	0.71
Time of post-C-CRT tooth extraction, mo. N (%) > 5.5 ≤ 5.5	61 (31.6) 132 (68.4)	18 (30.0) 42 (70.0)	43 (32.3) 90 (67.7)	0.82
ORN, N (%) Absent Present	172 (89.1) 21 (10.9)	42 (70.0) 18 (30.0)	130 (97.7) 3 (2.3)	< 0.001
ORN stage *0 1 2	172 (89.1) 16 (8.3) 5 (2.6)	42 (70.0) 13 (21.7) 5 (8.3)	130 (97.7) 3 (2.3) 0 (0.0)	< 0.001
Median C-CRT to ORN interval, mo. (range)	20.8 (12.4–27.6)	22.4 (12.4–27.6)	19.1 (11.8–26.4)	0.49
Concurrent chemotherapy cycles, N (%) 1 2–3	32 (16.6) 161 (83.4)	10 (16.7) 50 (83.3)	22 (16.5) 111 (83.5)	0.92

* Stage 0 denotes for absence of ORN

*Abbreviations: C-CRT* concurrent chemoradiotherapy, *HPR* hemoglobin-to-platelet ratio, *Gy* Gray, *MMD* mean mandibular dose, *MMPD* maximum mandibular point dose, *mo* months, *ORN* osteoradionecrosis, *V64* volume receiving ≥ 64 Gy

**Fig. 2:**
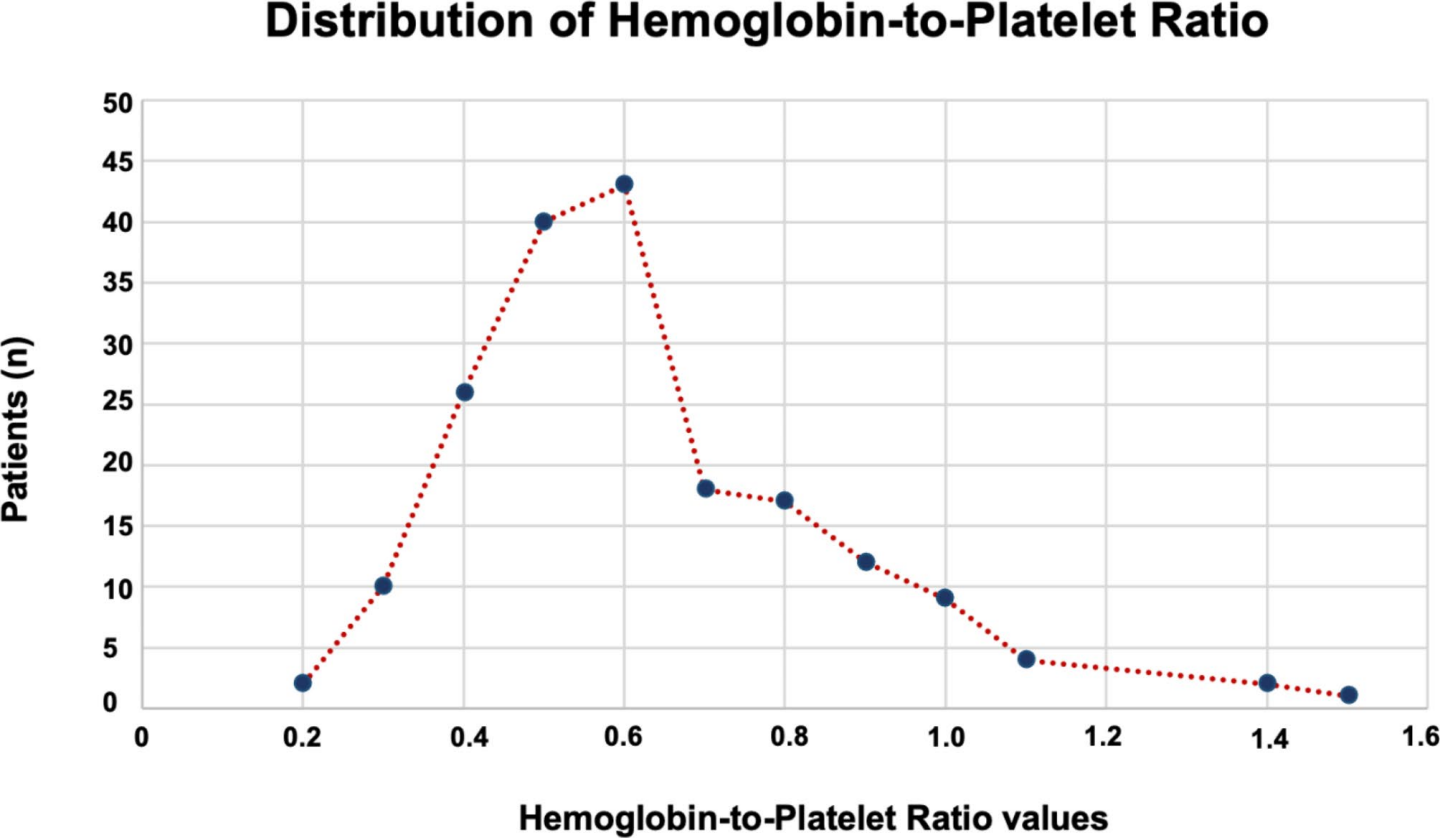
Distribution of hemoglobin-to-platelet ratio of all patients

**Fig. 3:**
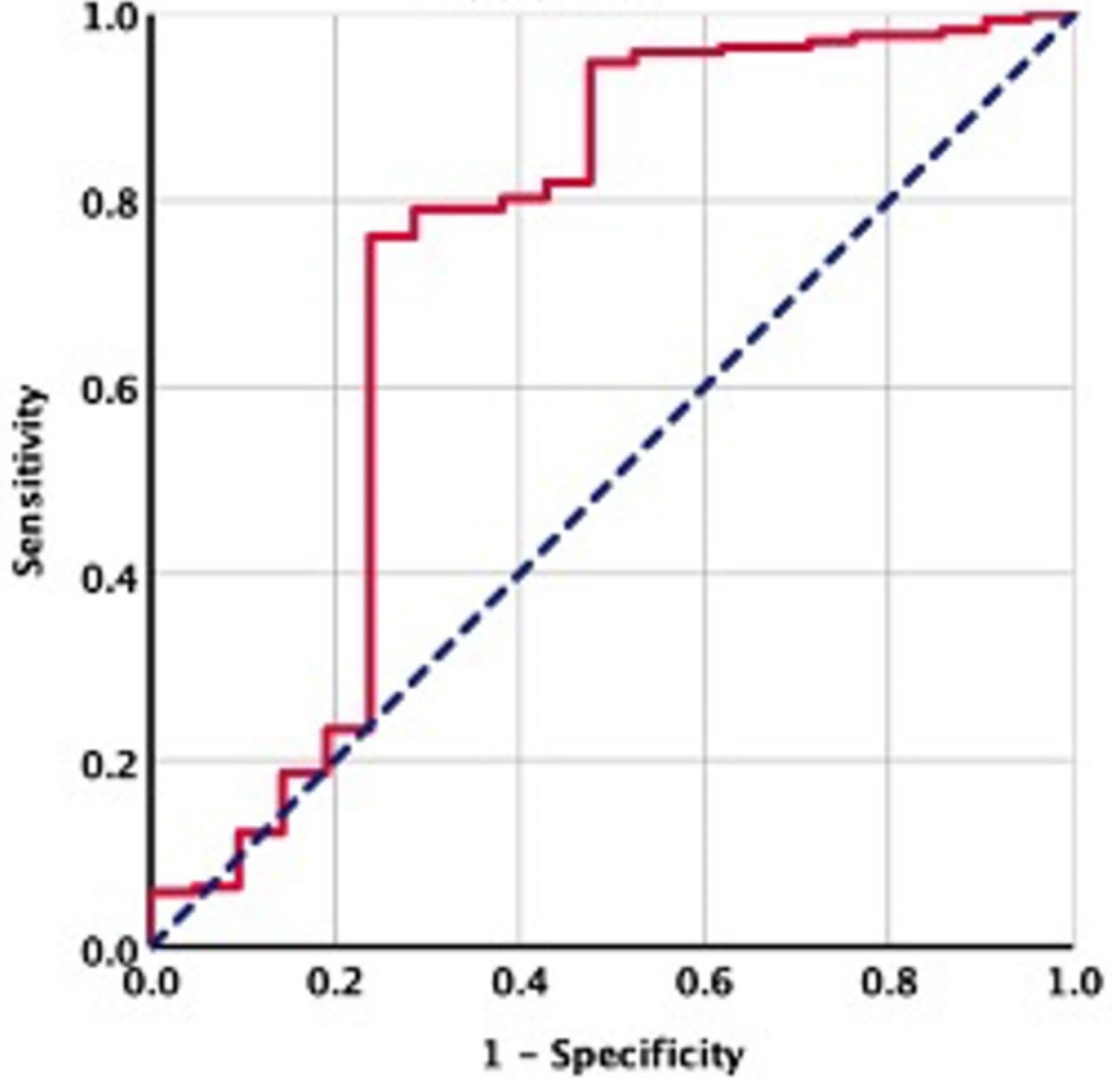
Results of the receiver-operating characteristic curve analysis evaluating the relationship between osteoradionecrosis prevalence after the concurrent chemoradiotherapy and the pretreatment hemoglobin-to-platelet ratio [Area under the curve (AUC): %73 sensitivity: 76.2%; and specificity: 71.4%]

Figure 2 depicts the distribution of pre-C-CRT HPR measures in all patients. We used ROC curve analysis to uncover a possible link between pre-C-CRT HPR levels and ORN rates, and the optimal cutoff point was 0.48 [area under the curve (AUC): 73%; sensitivity: 76.2%; and specificity: 71.4%] (Fig. 3), which divided the research population into two groups with significantly different ORN rates Group 1: HPR ≤ 0.48 (N = 60) and Group 2: HPR > 0.48 (N = 133), respectively. Except for the fact that 5 tooth extractions were significantly more prevalent in the HPR ≤ 0.48 group (45% vs. 19.5% for the HPR > 0.48 group; r_s_: -0.78; P < 0.001), all other pretreatment characteristics were nearly equally distributed between the two HPR groups (Table 1). Comparisons between the two groups revealed a significantly higher post-C-CRT ORN incidence rate in the HPR ≤ 0.48 group (30.0% vs. 2.3% for HPR > 0.48; P < 0.001) than in the HPR > 0.48 group. Furthermore, per Notani’s ORN staging, a pretreatment HPR ≤ 0.48 value was found to be significantly associated with more advanced ORN (stage 2) when compared to an HPR > 0.48 (8.3% vs. 0.0%; P < 0.001), implying that all ORNs in the HPR > 0.48 were stage 1 (Table 2).

**Fig. 4:**
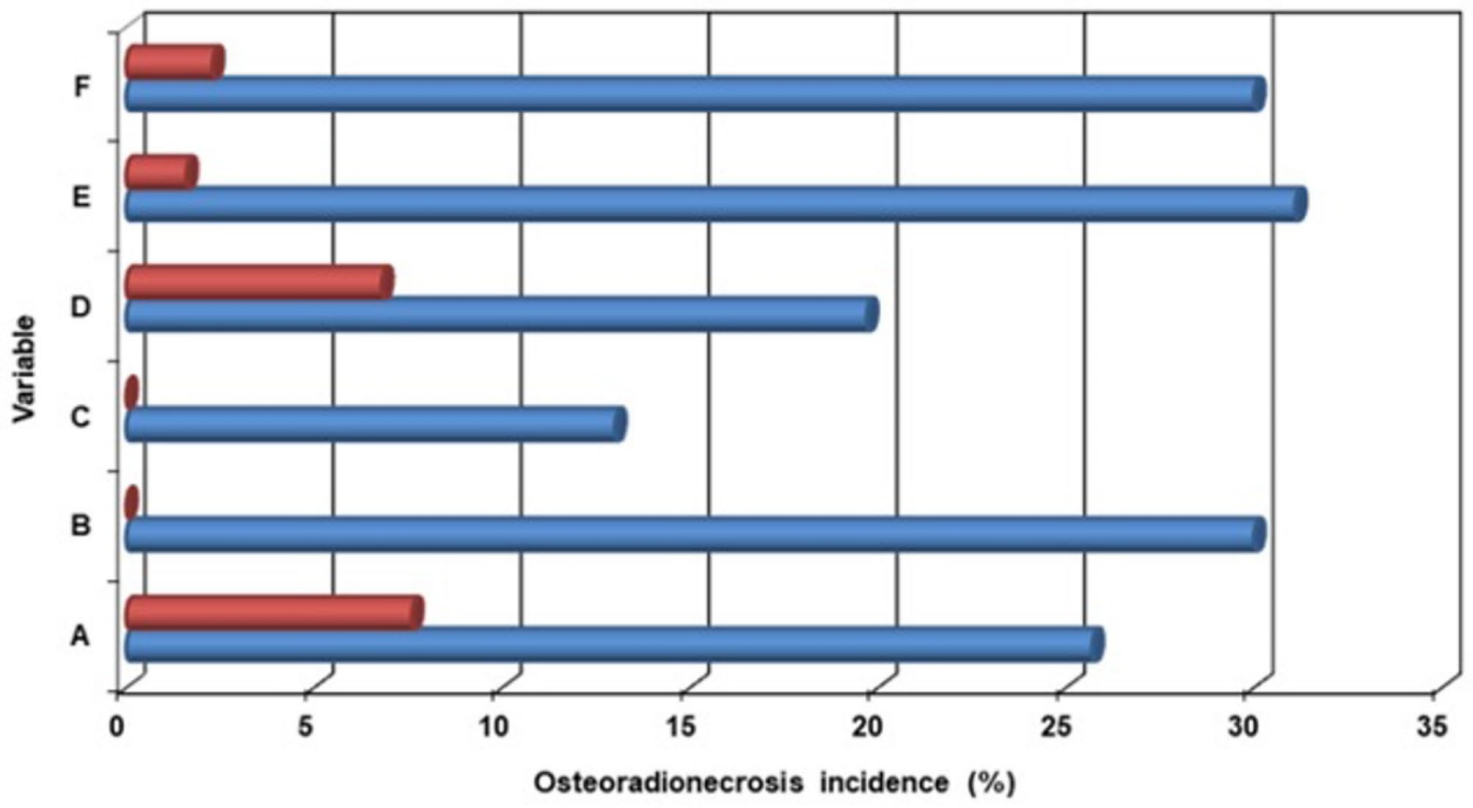
The bar graph demonstrates the incidence of osteoradionecrosis according to factors that were positive in multivariate analyzes. *Note*: A; V64 Gy, red bar: < 27%, blue bar: ≥ 27%, B; Pre-C-CRT tooth extraction, red bar: < 5, blue bar: ≥ 5, C; Post-C-CRT tooth extraction, red bar: Absent, blue bar: Present, D; MMD, red bar: < 50.6 Gy, blue bar: ≥ 50.6 Gy, E; C-CRT to tooth extraction interval, red bar: > 5.5 mo., blue bar: ≤ 5.5 mo., F; HPR, red bar: ≤ 0.48, blue bar: > 0.48. *Abbreviations: C-CRT* concurrent chemoradiotherapy, *Gy* Gray, *HPR* hemoglobin-to-platelet ratio, *MMD* mean mandibular dose, *mo* months, *ORN* osteoradionecrosis, *V64* mandibular volume receiving ≥ 64 Gy

The results of the univariate analysis revealed that, in addition to the HPR ≤ 0.48, a mandibular V64 ≥ 27% Gy (P = 0.002), pre-C-CRT ≥ 5 tooth extractions (P < 0.001), presence of post-C-CRT ≥ 1 tooth extractions (P < 0.001), MMD ≥ 50.6 Gy (P = 0.006), and a C-CRT to tooth extraction interval > 5.5 months (P < 0.001) were the other adverse factors linked to a higher ORN incidence rate (Table 3; Fig. 4). The findings of the multivariate logistic regression analysis indicated that all six variables retained their independent significance in terms of their impact on the post-C-CRT ORN incidence rates (P < 0.05 for each) (Table 3). Further analyses were carried out to look for possible correlations between dosimetric variables, but none were found between V64, MMPD, or MMD.

**Table 3:** Outcomes of multivariate analysis

Endpoint	All patients (N = 193)	ORN* (N = 21)	*P-*value	Odds ratio (95% CI)
V64 Gy, N (%) < 27% ≥ 27%	158 (81.9) 35 (18.1)	12 (7.6) 9 (25.7)	0.005	3.67 (2.18–6.43)
Pre-C-CRT tooth extraction, N (%) < 5 ≥ 5	123 (63.7) 70 (36.3)	0 (0.0) 21 (30.0)	< 0.001	11.7 (5.64–18.9)
Post-C-CRT tooth extraction, N (%) Absent Present	31 (16.1) 162 (83.9)	0 (0.0) 21 (13.0)	0.005	11.36 (4.72–18.67)
MMD, N (%) < 50.6 Gy ≥ 50.6 Gy	132 (68.4) 61 (31.6)	9 (6.8) 12 (19.7)	0.012	3.17 (2.56–5.18)
C-CRT to tooth extraction interval, N (%) > 5.5 mo. ≤ 5.5 mo.	61 (31.6) 132 (68.4)	19 (31.1) 2 (1.6)	< 0.001	7.89 (5.72–16.33)
HPR, N (%) ≤ 0.48 > 0.48	60 (31.1) 133 (68.9)	18 (30.0) 3 (2.3)	< 0.001	9.87 (3.16–20.78)

*ORN percentages reflect the rates in the related endpoint group

*Abbreviations: C-CRT* concurrent chemoradiotherapy, *CI* confidence interval, *Gy* Gray, *HPR* hemoglobin-to-platelet ratio,, *MMD* mean mandibular dose, *mo* months, *ORN* osteoradionecrosis, *V64* mandibular volume receiving ≥ 64 Gy

## Discussion

The primary purpose of the current research was to scrutinize whether the pre-C-CRT HPR values might predict post-C-CRT ORN incidence rates in LA-NPC patients who had undergone radical treatment. Our discoveries indicated a vigorous connection between low pre-C-CRT HPR levels (≤ 0.48) and significantly expanded ORN rates in this patient group (30.0% vs. 2.3% for HPR > 0.48; P < 0.001). The presence of pre-C-CRT ≥ 5 (P < 0.001) or post-C-CRT any number of teeth extraction history (P = 0.005), a C-CRT to tooth extraction interval > 5.5 months (P < 0.001), a mandibular V64 ≥ 27% Gy (P = 0.005), and MMD ≥ 50.6 Gy (P = 0.012) was likewise demonstrated to be connected to higher ORN occurrence rates.

The predictive expediency of novel biomarkers such as HPR, which reflect hypoxia, impaired vascular patency, exacerbated systemic and/or local inflammation, and suppressed immunity, has yet to be determined in the context of post-treatment ORN. Yet, the increased incidence of ORN after RT or C-CRT for head and neck cancers has been linked to various risk factors. Tobacco and alcohol abuse, pre- and post-RT tooth extractions, poor oral health or care, primary tumor type, advanced T-stage, advanced N-stage, use of C-CRT rather than RT, higher MMD, the volume of the mandible receiving higher RT doses, and use of non-IMRT techniques represent the most commonly cited risk factors [16, 45–48]. In this regard, we discovered that the V64 ≥ 27% Gy (P = 0.005), pre-C-CRT tooth extraction ≥ 5 (P < 0.001), post-C-CRT tooth extraction (P < 0.001), MMD ≥ 50.6 Gy (P = 0.012), C-CRT to tooth extraction interval > 5.5 months (P < 0.001), and a pre-C-CRT HPR ≤ 0.48 were independent drivers of higher ORN incidence rates in our research cohort. It is well-recognized that post-RT tooth extractions raise the risk of ORN more than pre-RT extractions, as do the use of C-CRT rather than RT and longer RT to tooth extraction intervals [48–51]. Higher MMD and larger mandibular volumes receiving higher RT doses are almost invariably linked to an increased incidence of ORN, which in our research was shown to be ≥ 50.6 and ≥ 27% for V64 Gy, respectively. These findings are consistent with a study by Moon et al., which found that MMD ≥ 49 Gy and V70 Gy ≥ 17% were significant risk factors for ORN development in a patient cohort consisting of oral cavity and oropharynx cancers [16].

Our study’s first novel finding was evidence of an explicit link between the number of pre-C-CRT tooth extractions and the likelihood of ORN after treatment. Despite the fact that the median interval between the extractions and IMRT commencement was at least 12 days (median: 17 days), patients who had ≥ 5 extractions had a remarkably higher risk of ORN than those who had < 5 extractions (30.0% vs. 0.0%; P < 0.001). Pretreatment tooth extractions have been linked to an elevated risk of post-RT ORN occurrence, which might be attributed to surgical trauma and its extent if multiple extractions are required, as in our study. Although this particular issue has never been thoroughly examined, the need for multiple tooth extractions may reflect a deteriorated oral health condition that contributes to ORN development [49]. Pre-RT tooth extractions do not preclude ORN development, according to Beech et al. [52]. Indeed, RT or C-CRT may facilitate the development of ORN in the rapidly remodeling bone of the healing socket by reducing tissue vascularity and oxygenation in such patients. A recent meta-analysis by Jiang et al. demonstrated that pre-RT tooth extractions have an ORN risk of 4.16%, which supports this announcement [50]. Another recent meta-analysis by Balermpas et al. indicated that extractions before IMRT were related to ORN development less frequently (8/432) than extractions after IMRT (7/92) (P < 0.01) [53]. This finding indicates that, even in the era of IMRT, tooth extractions preceding RT can reduce the frequency of ORN but not eliminate it. Unlike ours, all of these studies focused solely on the presence or absence of pretreatment tooth extractions, with no regard for the number of teeth extracted or the resulting trauma. As a result, albeit further research results are required to draw more reliable conclusions, the outcomes introduced here convincingly recommend a solid connection between the magnitude of jaw trauma and post-C-CRT ORN rates.

The most striking finding of the present cohort analysis was the demonstration of an independent association between a low pretreatment HPR value and an elevated ORN incidence in LA-NPC patients who received C-CRT. ORN incidence was significantly higher in the HPR ≤ 0.48 group than its HPR > 0.48 counterpart (30.0% vs. 2.3%; P < 0.001), suggesting that HPR might be a valuable biomarker for predicting ORN risk in these patients following C-CRT. It is difficult to characterize the specific processes behind this link because of the lack of basic or clinical studies addressing this relationship. Anyway, the critical functions of hemoglobin and platelets on tissue oxygenation, vascular patency, fibroatrophic processes, and immune and inflammation responses allow us to infer some logical hypotheses. Although bone lysis first manifests by a decrease in osteoblast counts as early as 4 h of RT, the primary driver of ORN seems to be radiation-induced endothelial injury and the following progressive vascular obliteration, microvascular damage, and bone destruction due to unmet oxygen and nutritional demands [54]. In any sort of tissue injury, expanded quantities of tissue oxygenation are required for every step of the healthy revitalization process, including cell proliferation, angiogenesis, collagen synthesis, epithelialization, and activation of bacterial defense mechanisms [5, 6]. Low hemoglobin levels may hamper bone repair processes during or after RT by worsening the existing hypoxic tissue conditions on the road to ORN formation. Such an unfavorable condition may further facilitate the ORN development by leading to the emergence of difficult to manage infections, exacerbated inflammation, and subsequent impairment of tissue repair, as most immune cells demand adequate oxygen levels to operate effectively [41]. Hypoxia induced secretion of HIF-1α and associated cytokines can generate and/or worsen systemic and localized inflammation, which can further deepen the hypoxic state and establish a vicious cycle of inflammation and hypoxia [34–36]. Given that low hemoglobin levels represent a state of systemic and localized hypoxia, all of these basic mechanisms, along with exacerbated hypoxia created by low hemoglobin levels, appear to support Marx’s theory of radiation-induced vasculature impairment, hypoxia, and impaired wound repair as the primary causes of ORN pathogenesis, though secondary infections may also play a role [27].

Higher platelet counts than required for adequate hemostasis may compromise blood flow in the inferior alveolar artery of the jaw bone, leading to vascular occlusion and hypoxia. This is due primarily to radiation-induced endarteritis, thrombosis, and the gradual obliteration of small arteries [14]. Uncontrolled radiation-induced thrombosis and ischemia inevitably result in cell loss and progressive fibrosis, as evidenced by avascular and hypo- or acellular bone marrow, bony lacunae with no osteocytes, endosteal atrophy with significantly reduced osteoblasts and osteoclasts, and periosteum fibrosis [33]. Radiation-induced thrombosis and ischemia can also cause an increase in the production of pro-inflammatory and inflammatory cytokines and chemokines, such as TNF-α, IL-1, and IL-6, which attract inflammatory cells to the wounded jaw. As a result of the aggravated local inflammation, some recruited monocytes may differentiate into M2 cells, which release TGF-ß, a key driver of radiation-induced fibrosis [42]. Furthermore, inflated TGF-ß secretion can also force myeloid progenitors to differentiate into myofibroblasts, which produce excessive quantities of proteoglycans, fibronectin, and collagen, provoking the injured tissues to stiffen and thicken [33, 42]. The presence of undue collagen inevitably reduces vascularity beyond tolerable limits, resulting in fibrotic tissue atrophy and necrosis. Such fibrotic regions are more susceptible to physical injuries and gradually increasing ischemia, which theoretically explains why patients with post-RT tooth extractions have a higher ORN rate [45]. Given the essence of platelets, as well as tissue oxygenation status, in a properly functioning immunity and inflammation response and tissue regeneration processes, the plausible mechanisms described in the last two paragraphs seem to lend support to the discovery of a strong relationship between a low pretreatment HPR value and an inflated incidence of ORN. Furthermore, our data imply that HPR and its components may play a critical role in ORN pathogenesis in all of Marx’s hypovascular and hypocellular [27], Delanian’s and Lefaix’s fibroatrophic [55], Lyons’ [56] and Bras et al.‘ fibroatrophy-related hypovascularity theories [29].

To clinically stratify patients into two risk groups, this study used a novel, simple to obtain and apply blood-borne biomarker. As a result, it can be used as a model for earlier identification of high-risk patients, allowing for stricter follow-up protocols, the use of more strict dosimetric constraints for the jaw, the prompt implementation of necessary oral hygiene and anti-infective measures, and the avoidance of invasive procedures like tooth extraction and dental implant placement to the greatest extent possible.

## Limitations

There were several limitations in the current study. First, the present findings represent the experiences of single institutional retrospective research with a relatively modest cohort size [57]. Second, our HPR cutoff, which relies upon single time-point measures, may not reflect the fittest HPR cutoff since HPR is a dynamic biomarker with striking time-dependent fluctuations. Therefore, future studies researching the HPR values acquired during or after the C-CRT spans may be beneficial in identifying a more suitable HPR cutoff that has a more unmistakable link with ORN. Third, none of the direct examinations of vascular abnormalities or in vivo oxygen measurements were available. Hence, blood flow and oxygen pressure measurements in the treatment area before, during, and after C-CRT may be beneficial in identifying individuals who are more likely to develop ORN. Consequently, the current findings should be interpreted with caution and viewed as hypothesis-generating rather than definitive guidance until large-scale prospective research can corroborate them.

## Conclusions

In conclusion, our retrospective cohort analysis outcomes indicated that a pretreatment HPR ≤ 0.48 was a strong predictor of significantly higher ORN incidence following C-CRT in definitely treated LA-NPC patients, which is a first in the literature. If confirmed, simple to achieve and calculate, affordable, and reproducible pretreatment HPR might serve usefully for dedicated risk stratification and the prompt enactment of screening, preventive, and therapeutic measures in such patients.

## Electronic supplementary material

Below is the link to the electronic supplementary material.

Supplementary Material 1

## Data Availability

Data cannot be shared publicly because the data is owned and saved by Baskent University Medical Faculty. Data are available from the Baskent University Radiation Oncology Institutional Data Access / Ethics Committee (contact via Baskent University Ethics Committee) for researchers who meet the criteria for access to confidential data: contact address, adanabaskent@baskent.edu.tr.

## Abbreviations

ADA: American Dental Association
AJCC: American Joint Cancer Committee
AUC: Area under the curve
C-CRT: Concurrent chemoradiotherapy
CT: Computed tomography
18-FDG-PET–CT: 18-fluorodeoxyglucose-positron emission tomography/computed tomography
FDA: US Food and Drug Administration
Gy: Gray
HIF-1: Hypoxia-induced factor-1
HPR: Hemoglobin-to-platelet ratio
IL: Interleukin
IMRT: Intensity-modulated RT
LA-NPC: Locally advanced nasopharyngeal carcinoma
M2: Macrophage
MMD: Mean mandibular dose
MMPD: Maximum mandibular point dose
N: Node
NPC: Nasopharyngeal carcinoma
ORN: Osteoradionecrosis
PTVs: Planning target volumes
ROC: Receiver operating characteristic
RT: Radiotherapy
SIB-IMRT: Simultaneous integrated boost intensity-modulated RT
T: Tumor
TGF-α, TGF-β: Transforming growth factor
TNF: Tumor necrosis factor
V64: Volume receiving ≥ 64 Gy
VEGF: Vascular endothelial growth factor.

## References

[1] Baujat B, Audry H, Bourhis J, Chan AT, Onat H, Chua DT (2006). Chemotherapy in locally advanced nasopharyngeal carcinoma: an individual patient data meta-analysis of eight randomized trials and 1753 patients. Int J Radiat Oncol Biol Phys.

[2] De Felice F, Musio D, Terenzi V, Valentini V, Cassoni A, Tombolini M (2014). Treatment improvement and better patient care: which is the most important one in oral cavity cancer?. Radiat Oncol.

[3] De Felice F, de Vincentiis M, Luzzi V, Magliulo G, Tombolini M, Ruoppolo G (2018). Late radiation-associated dysphagia in head and neck cancer patients: evidence, research and management. Oral Oncol.

[4] Pignon JP, le Maître A, Maillard E, Bourhis J, MACH-NC Collaborative Group (2009). Meta-analysis of chemotherapy in head and neck cancer (MACH-NC): an update on 93 randomised trials and 17,346 patients. Radiother Oncol.

[5] Meleca JB, Zhang E, Fritz MA, Ciolek PJ (2021). Overview and emerging Trends in the treatment of osteoradionecrosis. Curr Treat Options Oncol.

[6] Chapchay K, Weinberger J, Eliashar R, Adler N (2019). Anterior Skull Base Reconstruction following ablative surgery for osteoradionecrosis: Case Report and Review of Literature. Ann Otol Rhinol Laryngol.

[7] Jawad H, Hodson NA, Nixon PJ (2015). A review of dental treatment of head and neck cancer patients, before, during and after radiotherapy: part 1. Br Dent J.

[8] Jawad H, Hodson NA, Nixon PJ (2015). A review of dental treatment of head and neck cancer patients, before, during and after radiotherapy: part 2. Br Dent J.

[9] Buglione M, Cavagnini R, Di Rosario F, Sottocornola L, Maddalo M, Vassalli L (2016). Oral toxicity management in head and neck cancer patients treated with chemotherapy and radiation: Dental pathologies and osteoradionecrosis (part 1) literature review and consensus statement. Crit Rev Oncol Hematol.

[10] Nooh N (2013). Dental implant survival in irradiated oral cancer patients: a systematic review of the literature. Int J Oral Maxillofac Implants.

[11] Chrcanovic BR, Albrektsson T, Wennerberg A (2016). Dental implants in irradiated versus nonirradiated patients: a meta-analysis. Head Neck.

[12] Epstein JB, Rea G, Wong FL, Spinelli J, Stevenson-Moore P (1987). Osteonecrosis: study of the relationship of dental extractions in patients receiving radiotherapy. Head Neck Surg.

[13] Cheng VS, Wang CC (1974). Osteoradionecrosis of the mandible resulting from external megavoltage radiation therapy. Radiology.

[14] Rankow RM, Weissman B (1971). Osteoradionecrosis of the mandible. Ann Otol Rhinol Laryngol.

[15] Morrish RB, Chan E, Silverman S, Meyer J, Fu KK, Greenspan D (1981). Osteonecrosis in patients irradiated for head and neck carcinoma. Cancer.

[16] Moon DH, Moon SH, Wang K, Weissler MC, Hackman TG, Zanation AM (2017). Incidence of, and risk factors for, mandibular osteoradionecrosis in patients with oral cavity and oropharynx cancers. Oral Oncol.

[17] Caparrotti F, Huang SH, Lu L, Bratman SV, Ringash J, Bayley A (2017). Osteoradionecrosis of the mandible in patients with oropharyngeal carcinoma treated with intensity-modulated radiotherapy. Cancer.

[18] Mirabile A, Numico G, Russi EG, Bossi P, Crippa F, Bacigalupo A (2015). Sepsis in head and neck cancer patients treated with chemotherapy and radiation: literature review and consensus. Crit Rev Oncol Hematol.

[19] Costantino PD, Friedman CD, Steinberg MJ (1995). Irradiated bone and its management. Otolaryngol Clin North Am.

[20] Oh HK, Chambers MS, Garden AS, Wong PF, Martin JW (2004). Risk of osteoradionecrosis after extraction of impacted third molars in irradiated head and neck cancer patients. J Oral Maxillofac Surg.

[21] Støre G, Boysen M (2000). Mandibular osteoradionecrosis: clinical behaviour and diagnostic aspects. Clin Otolaryngol Allied Sci.

[22] Morrish RB, Chan E, Silverman S, Meyer J, Fu KK, Greenspan D (1981). Osteonecrosis in patients irradiated for head and neck carcinoma. Cancer.

[23] Horiot JC, Bone MC, Ibrahim E, Castro JR (1981). Systematic dental management in head and neck irradiation. Int J Radiat Oncol Biol Phys.

[24] Marx RE, Johnson RP (1987). Studies in the radiobiology of osteoradionecrosis and their clinical significance. Oral Surg Oral Med Oral Pathol.

[25] Thorn JJ, Hansen HS, Specht L, Bastholt L (2000). Osteoradionecrosis of the jaws: clinical characteristics and relation to the field of irradiation. J Oral Maxillofac Surg.

[26] Dumoulin S, van Maanen A, Magremanne M (2021). Dental prevention of maxillo-mandibular osteoradionecrosis: a ten-year retrospective study. J Stomatol Oral Maxillofac Surg.

[27] Marx RE (1983). Osteoradionecrosis: a new concept of its pathophysiology. J Oral Maxillofac Surg.

[28] Madrid C, Abarca M, Bouferrache K (2010). Osteoradionecrosis: an update. Oral Oncol.

[29] Bras J, de Jonge HK, van Merkesteyn JP (1990). Osteoradionecrosis of the mandible: pathogenesis. Am J Otolaryngol.

[30] Mo CJ, Hu ZJ, Qin SZ, Chen HP, Huang L, Li S (2020). Diagnostic value of platelet-lymphocyte ratio and hemoglobin-platelet ratio in patients with rectal cancer. J Clin Lab Anal.

[31] Helvaci MR, Aydogan A, Akkucuk S, Oruc C, Ugur M (2014). Sickle cell diseases and ileus. Int J Clin Exp Med.

[32] Darby IA, Hewitson TD (2016). Hypoxia in tissue repair and fibrosis. Cell Tissue Res.

[33] Davì G, Patrono C (2007). Platelet activation and atherothrombosis. N Engl J Med.

[34] Castillo-Rodríguez RA, Trejo-Solís C, Cabrera-Cano A, Gómez-Manzo S, Dávila-Borja VM (2022). Hypoxia as a modulator of inflammation and immune response in cancer. Cancers (Basel).

[35] Dhanda J, Pasquier D, Newman L, Shaw R (2016). Current concepts in Osteoradionecrosis after Head and Neck Radiotherapy. Clin Oncol (R Coll Radiol).

[36] Chrcanovic BR, Reher P, Sousa AA, Harris M (2010). Osteoradionecrosis of the jaws–a current overview–part 1: physiopathology and risk and predisposing factors. Oral Maxillofac Surg.

[37] Albisinni S, Pretot D, Al Hajj Obeid W, Aoun F, Quackels T, Peltier A (2019). The impact of neutrophil-to-lymphocyte, platelet-to-lymphocyte and haemoglobin-to-platelet ratio on localised renal cell carcinoma oncologic outcomes. Prog Urol.

[38] Beresford MJ, Burcombe R, Ah-See ML, Stott D, Makris A (2006). Pre-treatment haemoglobin levels and the prediction of response to neoadjuvant chemotherapy in breast cancer. Clin Oncol (R Coll Radiol).

[39] Zheng YY, Wu TT, Chen Y, Hou XG, Yang Y, Zhang JY (2020). Platelet-to-hemoglobin ratio as a novel predictor of long-term adverse outcomes in patients after percutaneous coronary intervention: a retrospective cohort study. Eur J Prev Cardiol.

[40] White SC, Pharoah MJ. Oral Radiology-E-Book: principles and interpretation. Elsevier: St. Louis, Missouri;; 2018. pp. 808–32.

[41] Yilmaz B, Somay E, Selek U, Topkan E (2021). Pretreatment systemic Immune-Inflammation Index Predict needs for Teeth Extractions for locally Advanced Head and Neck Cancer Patients undergoing concurrent Chemoradiotherapy. Ther Clin Risk Manag.

[42] Notani K, Yamazaki Y, Kitada H, Sakakibara N, Fukuda H, Omori K (2003). Management of mandibular osteoradionecrosis corresponding to the severity of osteoradionecrosis and the method of radiotherapy. Head Neck.

[43] Caponio VCA, Troiano G, Togni L, Zhurakivska K, Santarelli A, Laino L (2023). Pattern and localization of perineural invasion predict poor survival in oral tongue carcinoma. Oral Dis.

[44] Heinze G, Dunkler D (2017). Five myths about variable selection. Transpl Int.

[45] Reuther T, Schuster T, Mende U, Kübler A (2003). Osteoradionecrosis of the jaws as a side effect of radiotherapy of head and neck tumour patients–a report of a thirty year retrospective review. Int J Oral Maxillofac Surg.

[46] Owosho AA, Tsai CJ, Lee RS, Freymiller H, Kadempour A, Varthis S (2017). The prevalence and risk factors associated with osteoradionecrosis of the jaw in oral and oropharyngeal cancer patients treated with intensity-modulated radiation therapy (IMRT): the Memorial Sloan Kettering Cancer Center experience. Oral Oncol.

[47] Aarup-Kristensen S, Hansen CR, Forner L, Brink C, Eriksen JG, Johansen J (2019). Osteoradionecrosis of the mandible after radiotherapy for head and neck cancer: risk factors and dose-volume correlations. Acta Oncol.

[48] Khoo SC, Nabil S, Fauzi AA, Yunus SSM, Ngeow WC, Ramli R (2021). Predictors of osteoradionecrosis following irradiated tooth extraction. Radiat Oncol.

[49] Wang TH, Liu CJ, Chao TF, Chen TJ, Hu YW (2017). Risk factors for and the role of dental extractions in osteoradionecrosis of the jaws: a national-based cohort study. Head Neck.

[50] Jiang Y, Zhu X, Qu S (2014). Incidence of osteoradionecrosis in patients who have undergone dental extraction prior to radiotherapy: a systematic review and meta-analysis. J Oral Maxillofac Surg Med Pathol.

[51] Nabil S, Samman N (2011). Incidence and prevention of osteoradionecrosis after dental extraction in irradiated patients: a systematic review. Int J Oral Maxillofac Surg.

[52] Beech NM, Porceddu S, Batstone MD (2017). Radiotherapy-associated dental extractions and osteoradionecrosis. Head Neck.

[53] Balermpas P, van Timmeren JE, Knierim DJ, Guckenberger M, Ciernik IF (2022). Dental extraction, intensity-modulated radiotherapy of head and neck cancer, and osteoradionecrosis: a systematic review and meta-analysis. Strahlenther Onkol.

[54] Xu J, Zheng Z, Fang D, Gao R, Liu Y, Fan ZP (2012). Early-stage pathogenic sequence of jaw osteoradionecrosis in vivo. J Dent Res.

[55] Delanian S, Lefaix JL (2004). The radiation-induced fibroatrophic process: therapeutic perspective via the antioxidant pathway. Radiother Oncol.

[56] Lyons A, Ghazali N (2008). Osteoradionecrosis of the jaws: current understanding of its pathophysiology and treatment. Br J Oral Maxillofac Surg.

[57] Russo D, Mariani P, Caponio VCA, Lo Russo L, Fiorillo L, Zhurakivska K (2021). Development and validation of prognostic models for oral squamous cell carcinoma: a systematic Review and Appraisal of the literature. Cancers (Basel).

