# Supplementary material for: The predictive value of pretreatment hemoglobin-to-platelet ratio on osteoradionecrosis incidence rates of locally advanced nasopharyngeal cancer patients managed with concurrent chemoradiotherapy
Source: BMC Oral Health. 2023 Apr 20;23:231. doi: 10.1186/s12903-023-02937-9 (PMC10116666; doi:10.1186/s12903-023-02937-9)
Supplement: Supplementary file 1 — Supplementary Material 1 [file 12903_2023_2937_MOESM1_ESM.docx]

**Research Article**

**Title: The Predictive Value of Pretreatment Hemoglobin-to-Platelet Ratio on Osteoradionecrosis Incidence Rates of Locally Advanced Nasopharyngeal Cancer Patients Managed with Concurrent Chemoradiotherapy**

Busra Yilmaz^1^

Efsun Somay^2^

Erkan Topkan^3*^

Ahmet Kucuk^4^

Berrin Pehlivan^5^

Ugur Selek^6^

^1^ Department of Oral and Maxillofacial Radiology, Faculty of Dentistry, Baskent University, Ankara, Turkey. E-mail: uzmdtbusrayilmaz@gmail.com

^2^ Department of Oral and Maxillofacial Surgery, Faculty of Dentistry, Baskent University, Ankara, Turkey. E-mail: efsuner@gmail.com

^3^ Department of Radiation Oncology, Faculty of Medicine, Baskent University, Adana, Turkey. E-mail: [docdretopkan@gmail.com](mailto:docdretopkan@gmail.com)

^4^ Department of Radiation Oncology, Mersin City Hospital, Mersin, Turkey. E-mail: drakucuk@hotmail.com

^5^ Department of Radiation Oncology, Bahcesehir University, Istanbul, Turkey. E-mail: berrinpehlivan@gmail.com

^6^ Department of Radiation Oncology, School of Medicine, Koc University, Istanbul, Turkey. E-mail:[ugurselek@yahoo.com](mailto:ugurselek@yahoo.com)

*** Corresponding Author:**

Erkan Topkan

Department of Radiation Oncology, Faculty of Medicine, Baskent University, Adana, 01120 Turkey,

E-mail address: docdretopkan@gmail.com

Phone: +90 533 7381069

Fax: +90 322 3444452

Postal address: Kazım Karabekir District, Gulhatmi Street 37/A, 01120 Yuregir/Adana
